# Maxillary Arch Dimensions in 6-Year-Old Cleft Children in Northern Finland: A Cross-Sectional Study

**DOI:** 10.3390/ijerph18147432

**Published:** 2021-07-12

**Authors:** Mirjami Corcoran, Saujanya Karki, Leena Ylikontiola, Riitta Lithovius, George K. Sándor, Virpi Harila

**Affiliations:** 1 Research Unit of Oral Health Sciences, University of Oulu, 90220 Oulu, Finland; saujanya.karki@oulu.fi (S.K.); leena.ylikontiola@oulu.fi (L.Y.); riitta.lithovius@gmail.com (R.L.); george.sandor@oulu.fi (G.K.S.); virpi.harila@oulu.fi (V.H.); 2 Medical Research Centre Oulu, Oulu University Hospital and University of Oulu, 90220 Oulu, Finland

**Keywords:** cleft lip, cleft palate, initial cleft severity, maxillary arch dimensions

## Abstract

The aim was to cross-sectionally examine the maxillary arch dimensions in 6-year-old children with cleft lip and/or palate and to compare them with the initial cleft sizes among patients with cleft palate. The study included 89 patients with clefts treated at the Oulu University Hospital. The subjects were divided into three groups: cleft palate, cleft lip, and cleft lip and palate. Study casts were scanned, and the maxillary arch dimensions were examined using a 3D program (3Shape Orthoanalyzer, Copenhagen, Denmark). The statistical methods Student’s *t*-test and one-way ANOVA were used to compare the means (SD) between the groups. Spearman’s correlation coefficient was used to determine the correlation between cleft severity and maxillary dimensions. A significant difference was found between different initial cleft sizes in terms of distance between the second deciduous molar and the first incisor on the right side. The intermolar width showed a negative correlation with the initial cleft size. The dimensions were shorter for clefts affecting the palate and largest for clefts affecting only the lip. Larger clefts resulted in a shorter maxilla on the right side. Many dimensions became shorter when the initial cleft was larger. Clefts of the palate resulted in smaller maxillas.

## 1. Introduction

Cleft lip and/or palate (CLP) are the most common congenital craniofacial anomalies [1]. The worldwide incidence has been estimated to be 1.0–2.1/1000 [2]. The incidence of clefts in Finland is higher than in other European countries, and it has been estimated to be 2.56 cases per 1000 live births and abortions. The incidence of cleft palate (CP) in Finland is one of the highest in the world [1].

Facial clefts can affect the palate, the lip, or both. Clefts can be unilateral or bilateral. Clefts can be divided to cleft palate (CP), cleft lip (CL), and cleft lip and palate (CLP). Children with clefts have been found to have retrognathic and narrow maxilla [3,4,5] and micrognathia of the mandible [6]. They often need orthodontic treatment to manage malocclusions [7,8]. The scar tissue formed after the repair of the cleft is thought to affect the growth of the maxilla [9,10]. Maxillary arch dimensions have previously been suggested to be smaller in unilateral cleft lip and palate (UCLP) and bilateral cleft lip and palate (BCLP) patients compared with cleft lip (CL) [11] and cleft palate (CP) patients [5,12,13], but contrary results suggest similar dimensions in UCLP and CP patients [14].

Previous studies regarding the association between the initial cleft size and maxillary outcome have reported varying results. No clear association has been found between cleft severity and maxillary arch dimensions in Finnish CP children [5]. Among Swedish CP children, the initial cleft size did not seem to affect the maxillary outcome either [15]. In turn, the maxilla has been reported to be shorter and more retrusive in Taiwanese UCLP patients with larger clefts [10], whereas a study among Australian UCLP patients did not find an association between those variables [16].

The prevalence of cleft palate in Finland is among the highest in the world [1]. The literature about CP patients is scarce, whereas many studies have examined patients with UCLP. The rare distribution of clefts in Northern Finland gives us an exceptional chance to examine CP patients closely. By investigating the relationship between the initial cleft size and maxillary arch dimensions among patients with CP, one can evaluate the importance of the initial cleft size as a possible factor affecting treatment outcome. The aim of the study was to compare the initial cleft size with maxillary arch dimensions among patients with CP at 6 years of age before the dental arches might be influenced by orthodontic treatment. Another aim was to examine the differences in maxillary arch dimensions between different cleft types and sexes. It was hypothesized that larger initial clefts and clefts including the palate result in smaller maxillary arch dimensions.

## 2. Materials and Methods

The documents of the participants were taken from routine follow-up examinations by the Oulu University Hospital cleft team in accordance with the Eurocleft program that the hospital follows [17]. The models were taken from all the cleft patients who had turned 6. At this age, the dental arches have not been influenced by orthodontics yet. No additional models were taken or examinations performed for this study. The hospital’s medical database was used for the collection of each participant’s medical history. The subjects of this retrospective study consisted of 89 6-year-old Finnish children born with cleft lip and/or palate and treated at the Oulu University Hospital (OUH) Cleft Lip and Palate Center in Northern Finland between 1996 and 2012. All the participants were treated by the same operators and techniques. The subjects received their regular dental care at their primary health-care centers. The closure of the lip was performed at approximately 3 months of age, and the closure of the palate at 9–12 months of age, depending on the severity of the cleft. Palates were repaired using either a straight-line closure for the most simple and least severe clefts or the von Langenbeck method for the moderate-sized clefts and included vomer flaps for the most severe clefts. For all of the palatal clefts, intravelar veloplasty was performed. This study was approved by the Northern Ostrobothnia Hospital District Ethical Committee (permission number 44/2019).

### 2.1. Initial Cleft Size

Classified grades based on the initial cleft size were used for the participants with CP in the study. Cleft severity was previously determined from three-dimensional scanned dental models by Lithovius and colleagues [18] by using a scale proposed by Jensen and colleagues [19]. Casts were taken before palatal closure. The extent of the overt cleft palate was divided into four grades increasing from smallest to largest (1–4): grade 1, soft palate; grade 2, less than one-third of the hard palate; grade 3, greater than one-third of the hard palate up to subtotal; and grade 4, total, cleft extending to the incisive foramen. The same grading has previously been used [20]. The operator estimated the cleft sizes from dental casts and pictures, if available. A total of 55 CP participants satisfied the inclusion criteria. The number of participants in the groups included 11 with grade 1, 14 with grade 2, 21 with grade 3, and 9 with grade 4 (Table 1).

### 2.2. Maxillary Arch Dimensions

The dental plaster casts used were taken with alginate impressions by the same operators of the cleft team. The casts were scanned using a surface laser scanner (3Shape R700^TM^ Scanner, 3Shape, Copenhagen, Denmark). The reference points and linear measurements were performed by one operator on the maxillary casts using 3Shape OrthoAnalyzer^TM^ software (3Shape A/S, Copenhagen, Denmark), which enables three-dimensional examination. The validation and reliability of the 3D method have previously been documented [21,22]. The measurements were performed by one operator to eliminate errors.

The measurements were made for both transverse and sagittal dimensions in the mixed dentition. In another study, the same maxillary dimensions were examined with the same 3D program and were shown to have good reliability [23].

**Figure 1:**
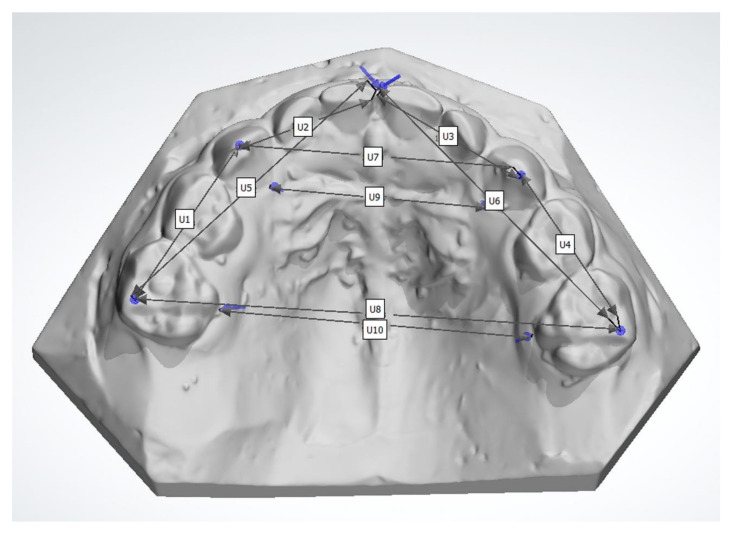
Maxillary arch dimensions.

The definition of the linear measurements used in this study are illustrated in Figure 1. The measurements included the distance between the maxillary second deciduous molars and the deciduous canines (U1 right side, U4 left side), the deciduous canines and the first incisors (U2 right side, U3 left side), the second deciduous molars and the first incisors (U5 right side, U6 left side), the inter-deciduous-canine width (U7), and inter-second-deciduous-molar width (U8), as well as the palatal width between the gingival part of the deciduous canines (U9) and the second deciduous molars (U10). The palatal measurement U9 was added to the previously used method [23] and, to our knowledge, has not been used before. The distances were measured to the nearest 0.01 mm. The distances with a tooth as a landmark were measured from the crown tip of the deciduous canines and the distobuccal cusp for the second deciduous molars. For the incisors, the reference point was the most mesial point of the tooth. The gingival reference points were measured from the most palatal point of the gingiva, measuring the shortest distance between the teeth.

The reliability and intraoperator reproducibility of the measurements were determined from randomized duplicate recordings. On a separate occasion, 20 randomly selected casts were remeasured. The intraclass correlation coefficient (ICC) for all the measurements was 0.996 (average measures) (single measures, 0.992). There was an excellent correlation of repeated measurements, indicating an excellent level of intra-operator reliability. No pre- or postsurgical orthodontic treatment was performed before the casts were taken prior to the palate closure and at the age of 6. Single dimensions that could not be measured due to incompletely modeled dental casts, inadequate eruption, or a missing tooth or landmark were excluded from the study.

### 2.3. Cleft Type

The subjects consisted of 6-year-old Finnish children born with a cleft lip and/or palate and treated at OUH between 1996 and 2012. The subjects represented all the following cleft types: isolated cleft palate, isolated cleft lip alone (uni- or bilateral), cleft lip and palate (uni- or bilateral), and submucous cleft palate. Participants with a submucous cleft palate and participants with missing medical records were excluded from the study.

The participants were divided into three groups based on their diagnoses: cleft palate CP, cleft lip CL, and cleft lip and palate CLP.

### 2.4. Statistical Analyses

All data were transferred into a database for analyses using the SPSS software (IBM SPSS Statistics for Windows, version 27.0, IBM Corp., Armonk, NY, USA). The statistical methods used included Student’s t-test, one-way ANOVA, and Spearman’s correlation coefficient. The intraclass correlation coefficient was used to examine the reliability of the method and operator. For the analyses, the participants were divided into three groups: cleft palate CP, cleft lip CL, and cleft lip and palate CLP. The means and standard deviation for the maxillary dimensions were calculated for the initial cleft size groups, different cleft types, and boys and girls. Spearman’s correlation test was used to determine the correlation between cleft severity and maxillary dimensions. The data were tested for normality with the Kolmogorov–Smirnov test and a histogram. The data represented a normal distribution, and therefore, parametric tests were used. One-way ANOVA was used to compare the means between cleft severity and maxillary arch dimensions and the means between cleft type and maxillary arch dimensions. Bonferroni correction was used as a post hoc analysis to determine the differences between groups. Student’s t-test was used to compare the maxillary arch dimensions between sexes. Pairwise deletion was used for missing data. Single missing data were deleted, but the existing data of the subjects were included in the analyses. For the analysis, *p < 0.05* was considered statistically significant.

## 3. Results

**Figure 2:**
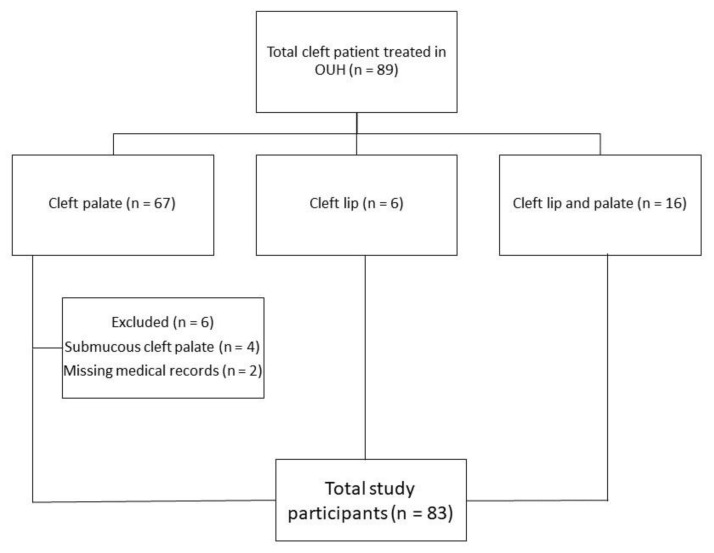
Study participants.

There were 67 participants with cleft palate (CP) (specifically, 63 participants with cleft palate and 4 participants with submucous cleft palate), 6 participants with isolated cleft lip (CL) (specifically, 1 participant with bilateral cleft lip, 5 participants with unilateral cleft lip), and 16 participants with cleft lip and palate (CLP) (specifically, 7 participants with bilateral cleft lip and palate and 9 participants with unilateral cleft lip and palate) (Table 1). Participants with submucous cleft palate (*n* = 4) and participants with missing medical records (*n* = 2) were excluded from the study. Thereafter, a total of 83 participants were included in the study (*n* = 83 vs. 89, 93.2%) (Figure 2).

The dental casts used for this study were taken at the mean age of 5.98 years (SD = 0.73). Girls slightly dominated the study population (*n* = 47 vs. *n* = 38). The most common cleft type in this study was cleft palate (*n* = 61). The number of missing data for each variable of interest was as follows: U1, *n* = 0; U2, *n* = 12; U3, *n* = 5; U4, *n* = 3; U5, *n* = 12; U6, *n* = 10; U7, *n* = 0; U8, *n* = 2; U9, *n* = 2; and U10, *n* = 4.

The maxillary arch dimensions of the subjects with CP were compared with the initial cleft size. The distance between the second deciduous molar and the first incisor on the right side (U5) was significantly shorter for the initial cleft size grade 4 compared with grades 2 and 3 (Table 2a).

When the maxillary dimensions were compared between the diagnosis groups (cleft types), the distance between the deciduous canine and the first incisor on the left side (U3) was significantly shorter for the CLP and CP groups compared with the CL group. Similar results were found for the inter-deciduous-canine width (U7) and the width between the most gingival points of the deciduous canines (U9) (Table 2a,b). However, the distance between the first incisor and the second deciduous molar on the left side (U6) was slightly broader in the CLP group compared with the CL group (Table 2b).

The sagittal distance between the second deciduous molar and the first incisor on the right side (U5) was significantly bigger for the boys compared with the girls. The same was true with respect to the transverse width between the second deciduous molars (U8) and the most gingival point of the second deciduous molars (U10) (Table 2a,b).

There was a negative correlation between U5 and the initial cleft size and also between U8 and the initial cleft size (Table 3).

## 4. Discussion

This study aimed to evaluate the maxillary arch dimensions among 6-year-old children with CLP in Northern Finland. The aim was to investigate the connection between maxillary arch dimensions and the initial cleft size among patients with CP and compare the maxillary arch dimensions between different cleft types and sexes. Larger clefts resulted in a significantly shorter sagittal distance between the second deciduous molar and the first incisor on the right side (U5), reflecting decreased anteroposterior growth. Clefts affecting the palate (CLP and CP) resulted in shorter sagittal and transverse maxillary dimensions than clefts affecting only the lip.

This study focused on comparing the maxillary arch dimensions between patients with clefts; thus no control group was included for evaluating the dimensions in noncleft children, which can be considered a weakness of this study. Another weakness of this study is the lack of a registered growth status. Other limitations are the cross-sectional nature of this study and the small sample size for some groups. However, our study comprised almost all the 6-year-old patients with clefts treated in Northern Finland since 1995.

The results of this study show no extensive significant connection between the initial cleft size and maxillary arch dimensions in patients with CP. One dimension (U5, between the second deciduous molar and the first incisor on the right side) out of 10 was significantly different for the cleft severity groups and was found between grade groups 2 and 4. In addition, one dimension (U8, inter-second-deciduous-molar width) showed a correlation with the initial cleft size but was not significant with other tests. Grade 2 patients showing multiple larger dimensions than grade 1 patients is contrary to our hypothesis. However, many dimensions were shorter when the initial cleft was larger, which supports our hypothesis. Clefts affecting the palate resulted in smaller maxillas as was hypothesized. The results confirmed the initial hypothesis. Studies with bigger sample sizes are needed to gain more statistical power.

The strength of this study is the consistent treatment our subjects received. All of them received their special medical care in the same hospital by the same professionals using the same techniques. The examination of the casts was always performed by the same operator, and the grading for the initial cleft size was performed by only one operator [18].

In previous studies, contrary results were reported regarding the association between cleft severity and maxillary arch dimensions. In a Swedish study, the initial cleft size did not affect the maxillary arches evaluated from the prevalence of crossbites in patients with CP and UCLP [15]. In a Finnish study, maxillary arch depth decreased slightly with the severity of the cleft, but no clear association was found between cleft severity and arch dimensions in 3-year-old children with CP [5]. No correlation was found between the initial cleft size and dental arch relationships in 6-year-old Australian patients with UCLP either [16], whereas contrary results showed a correlation between the cleft size and maxillary growth in patients with UCLP, measured from cephalometric radiographs [10]. In the aforementioned study, the maxilla was shorter and more retrusive in patients with larger clefts. Additionally, a systematic review revealed contradictory results about the connection between cleft severity and maxillary growth in patients with UCLP [24].

A Finnish study comparing 6-year-old female patients with submucous cleft palate (SMCP) and isolated cleft palate found no skeletal difference between them using lateral cephalograms. Only minor growth disturbance causing slight skeletal retrusion of the maxilla and mandible was found in both groups. Since submucous cleft palate is a milder form of overt cleft palate, the authors suggest that skeletal development may be associated with clefting per se [25]. This supports the results of the present study that show no extensive connection between cleft severity and maxillary arch dimensions at 6 years of age. In this study, subjects with submucous cleft palate were excluded (*n* = 4), but it can be deduced that the morphology of these patients would be similar to that of Heliövaara’s study subjects with submucous cleft palate [25]. According to the literature and the results of this study, no distinct conclusions can be made about the relationship between the initial cleft size and maxillary arch dimensions. However, the results show a trend of the larger initial clefts resulting in smaller maxillas. Studies with bigger sample sizes are needed to clarify this connection. It must be noted that the literature about patients with CP is scarce. CP and UCLP are different conditions, and studies about them should be compared with caution.

The findings of this study suggesting that clefts affecting the palate (UCLP, BCLP, CP) have smaller dimensions than clefts affecting only the lip are supported by previous studies. A Brazilian study examined the transversal dimensions of the maxillas of 5-year-old cleft children and concluded that clefts affecting the palate have more maxillary dimensional alterations than those without cleft palate [11]. A study among 6-year-old patients with clefts [13] and another one among 3-year-olds [5] both reported that BCLP have the narrowest maxillary dimensions and CL has the widest. Patients with UCLP have been reported to have smaller maxillary arch dimensions than patients with CP [5,12], which also supports our findings. A study among Northern Finland patients with clefts using the GOSLON Yardstick method showed that CL and SMCP all have good prognosis based on dental arch relationship. Most patients with CP had good prognosis (84.6%), followed by UCLP (66.7%) and BCLP (40%) [26]. The GOSLON Yardstick study highlights the prognosis of different cleft types and is in agreement with the findings of this study since the maxillary dimensions showed a similar trend between cleft types. However, results suggesting similar maxillary arch dimensions between patients with UCLP and CP at 5 years of age have been reported [14].

The results of the present study support those of previous studies, which suggest that patients with cleft have differences in maxillary arch dimensions between boys and girls [3,13]. Laitinen and colleagues [13] suggested that boys have larger maxillary dimensions at 6 years of age. The results of this study show that boys have a wider maxilla transversally since they have a significant difference in inter-second-molar width (from the cuspid and gingival points, U8 and U10). This has been reported previously too, albeit in patients with UCLP [3]. This study also found a difference between boys and girls in terms of distance between the second deciduous molar and the incisor on the right side (U5), reflecting boys having a longer maxilla on the right side. The study population was dominated by girls, and this might affect the results. Overall, most of the dimensions (70%) in this study were not significantly bigger for boys.

Maxillary dimensions have previously been examined using different and fewer parameters than ours. Some of the methods used in previous studies are Moore’s method [3,5,13] and the GOSLON Yardstick method [16,26]. Previous studies measured dental casts with digital calipers or used X-ray imaging. One study measured scanned images of the dental casts with digital calipers using a digital imaging software program [3], and another one studied cleft severity from scanned images of the dental casts using an image analyzing program. [16]. The timing of the evaluation of the initial cleft differed between studies. Some measured it at the time of the cleft closure [9,15], and some neonatally [5,16]. In this study, the casts were taken before the closure of the cleft. However, the timing for the evaluation of the initial cleft is assumed not to have major importance.

Traditionally, dental casts are used to measure dental arch dimensions. To our knowledge, this is the first study using 10 different sagittal and transverse dimensions measured from 3D casts of the maxilla (Figure 1). In this study, a 3D program was used that enables three-dimensional examination and magnification of the scanned casts. A three-dimensional technique decreases measuring errors as well. More studies with advanced 3D techniques would help compare dimensions and evaluate the new technology in examining maxillary arch dimensions among patients with cleft. Our intraclass correlation coefficient showed excellent correlation of repeated measurements, which demonstrates the high reliability of this technique. Similar results were reported in previous studies also [21,22,23].

Interestingly, this study showed no extensive connection between the initial cleft size and maxillary arch dimensions at 6 years of age. It was expected that children with larger initial clefts would have smaller maxillary arch dimensions at 6 years of age as a result of a deficient maxillary growth. However, only 1 parameter (U5) out of 10 showed a significant difference between the initial cleft size groups. In this study population, initial cleft size seems to significantly affect only the sagittal (between the first incisor and the second deciduous molar) growth of the maxilla on the right side. However, a trend of larger initial clefts resulting in smaller maxillas can be seen. The transversal distance between the second deciduous molars showed a correlation with the initial cleft size too. Clinically, smaller maxillas could appear as crowding and crossbites. The clinical significance impinges on the treatment planning, expected treatment outcome, and retention techniques after the orthodontic treatment. Additionally, orthognathic surgeries might be needed if the treatment outcome cannot be reached with orthodontic treatment. Future studies should focus on the possible clinical outcomes of these findings. The initial cleft size among patients with CP has previously been suggested to not have an association with the prevalence of crossbite at the age of 5 [15]. A high prevalence (62%) of anterior crossbites was found among 6-year-old patients with unilateral cleft lip and palate (UCLP), but no association was examined between the crossbites and cleft severity [8]. The study found no significant association between transversal maxillary dimensions or anterior crossbites and a later need for orthognathic surgery [8]. The growth of the maxilla is multifactorial, and there might not be a one-way relationship between the variables measured in the present study.

The results of this study improve the understanding of the relationship between initial cleft morphology and the outcome evaluated from the maxillary arch dimensions. The severity of the cleft does seem to play a role in the outcome of patients with CP, although we lack extensive evidence of a connection between cleft severity and maxillary growth. However, maxillary growth is reduced among clefts affecting the palate per se, and this should be noted when evaluating the outcome. The study results should be confirmed by studies with larger sample sizes.

## 5. Conclusions

We conclude that in the Northern Finnish cleft palate population, larger initial clefts result in a shorter maxilla on the right side at 6 years of age. Clefts affecting the palate result in smaller maxillary arch dimensions than clefts affecting only the lip. For boys with clefting, the maxilla is longer on the right side and wider posteriorly compared with girls. The results of this study cannot be generalized to other study populations.

## Figures and Tables

**Table 1:** Characteristics of the study participants.

	Number of Participants (Proportion)	Total
Gender		85
Boys	30(35.3%)	
Girls	55(64.7%)	
Cleft severity (CP patients)		55
Grade 1	11(20.0%	
Grade 2	14(25.5%)	
Grade 3	21(38.2%)	
Grade 4	9(16.4%)	
Cleft type		85
CP	63 (74.1%)	
CL	6 (7.0%)	
CLP	16 (18.8%)	

CP: cleft palate; CL: cleft lip; CLP: cleft lip and palate.

**Table 2:** (**a**) Number of participants (*n*) and means (SD) of the maxillary arch dimensions (U1–U5) by gender, cleft severity, and cleft type. (**b**) Number of participants (*n*) and means (SD) of the maxillary arch dimensions (U6–U10) by gender, cleft severity, and cleft type.

**(a)**										
**Characteristics**	***n*/Mean (SD) U1**	***p*-Value**	***n*/Mean (SD) U2**	***p*-Value**	***n*/Mean (SD) U3**	***p*-Value**	***n*/Mean (SD) U4**	***p*-Value**	***n*/Mean (SD) U5**	***p*-Value**
Gender										
Boys	38/16.82 (1.29)	0.400 ^a^	33/14.85 (1.72)	0.699 ^a^	32/14.56 (3.70)	0.410 ^a^	36/16.61 (1.44)	0.223 ^a^	33/29.98 (1.82)	0.015 *^,a^
Girls	45/16.61 (0.99)		38/14.70 (1.53)		45/15.17 (2.18)		44/16.07 (1.74)		38/28.90 (1.83)	
Cleft severity										
Grade 1	11/16.76 (1.26)	0.472 ^b^	9/14.48 (0.97)	0.110 ^b^	9/14.57 (1.57)	0.834 ^b^	11/16.70 (1.27)	0.509 ^b^	9/29.67 (1.59)	0.005 *^,b^
Grade 2	14/17.00 (0.71)		13/15.00 (0.89)		13/15.17 (1.09)		14/16.25 (1.71)		13/30.15 (1.14)	
Grade 3	21/16.58 (0.97)		17/15.07 (1.07)		18/15.07 (1.43)		21/16.54 (0.87)		17/29.68 (1.28)	
Grade 4	8/16.35 (1.17)		7/13.92 (1.68)		9/14.93 (2.33)		8/15.59 (3.34)		8/29.20 (0.75)	
Cleft type										
CP	61/16.74 (1.06)	0.600 ^b^	52/14.82 (1.28)	0.234 ^b^	56/15.11 (1.62)	<0.001 **^,b^	61/16.30 (1.64)	0.249 ^b^	52/29.35 (1.72)	0.903 ^b^
CL	6/16.25 (1.62)		5/15.64 (3.23)		6/18.09 (3.76)		5/15.13 (2.09)		5/29.38 (4.02)	
CLP	16/16.75 (1.23)		14/14.26 (1.93)		15/12.92 (4.64)		14/16.50 (1.26)		14/29.61 (1.64)	
**(b)**										
**Characteristics**	***n*/Mean (SD) U6**	***p*** **-Value**	***n*/Mean (SD) U7**	***p*** **-Value**	***n*/Mean (SD) U8**	***p*** **-Value**	***n*/Mean (SD) U9**	***p*** **-Value**	***n*/Mean (SD) U10**	***p*** **-Value**
Gender										
Boys	31/29.64 (3.09)	0.842 ^a^	37/27.61 (4.20)	0.719 ^a^	37/43.75 (3.11)	0.006 *^,a^	37/21.60 (3.41)	0.794 ^a^	36/28.45 (2.78)	0.002 *^,a^
Girls	42/29.52 (1.83)		46/27.89 (2.30)		44/42.03 (2.05)		44 /21.43 (2.16)		43/26.58 (2.40)	
Cleft severity										
Grade 1	9/30.06 (2.17)	0.300 ^b^	11/27.39 (1.57)	0.246 ^b^	11/43.08 (2.85)	0.071 ^b^	10/21.48 (1.30)	0.579 ^b^	10/27.12 (2.61)	0.205 ^b^
Grade 2	13/30.38 (1.41)		14/28.24 (1.21)		14/43.66 (1.80)		14/22.07 (1.79)		13/27.71 (2.29)	
Grade 3	18/29.61 (1.40)		21/21.33 (2.50)		21/43.17 (2.48)		21/22.11 (2.39)		21/28.07 (2.95)	
Grade 4	8/29.20 (0.75)		8/29.20 (1.16)		8/41.00 (1.36)		8/21.14 (1.50)		8/25.82 (1.60)	
Cleft type										
CP	54/28.04 (1.95)	0.003 *^.b^	62/29.04 (1.95)	<0.001 **^,b^	61/42.88 (2.50)	0.446 ^b^	60/21.80 (1.93)	<0.001 **^,b^	59/27.43 (2.72)	0.449 ^b^
CL	5/29.38 (4.02)		6/32.48 (1.38)		5/43.90 (3.19)		6/24.46 (2.22)		6/28.62 (1.25)	
CLP	14/29.61 (1.64)		15/24.74 (5.06)		15/42.19 (3.40)		15/19.12 (4.08)		14/26.92 (3.19)	

CP: cleft palate; CL: cleft lip; CLP: cleft lip and palate. * *p*-Value < 0.05; ** *p*-value < 0.001. ^a^ *p*-Value calculated using Student’s *t*-test. ^b^ *p*-Value computed using one-way ANOVA.

**Table 3:** Correlation between maxillary arch dimensions and cleft severity.

	Cleft Severity (1–4)	
	Rho	*p*-Value
U1	−0.187	0.176
U2	−0.072	0.635
U3	−0.051	0.730
U4	−0.020	0.885
U5	−0.338	0.020 *
U6	−0.215	0.142
U7	−0.068	0.623
U8	−0.284	0.038 *
U9	−0.134	0.245
U10	−0.086	0.541

* *p*-Value < 0.05. *p*-Value computed using Spearman’s correlation coefficient.
